# Two overlapping two-component systems in *Xanthomonas oryzae* pv. *oryzae* contribute to full fitness in rice by regulating virulence factors expression

**DOI:** 10.1038/srep22768

**Published:** 2016-03-09

**Authors:** Dehong Zheng, Xiaoyan Yao, Meng Duan, Yufeng Luo, Biao Liu, Pengyuan Qi, Ming Sun, Lifang Ruan

**Affiliations:** 1 State Key Laboratory of Agricultural Microbiology, College of Life Science and Technology, Huazhong Agricultural University, Wuhan 430070, PR China

## Abstract

Two-component signal transduction systems (TCSs) are widely used by bacteria to adapt to the environment. In the present study, StoS (stress tolerance-related oxygen sensor) and SreKRS (salt response kinase, regulator, and sensor) were found to positively regulate extracellular polysaccharide (EPS) production and swarming in the rice pathogen *Xanthomonas oryzae* pv. *oryzae* (*Xoo*). Surprisingly, the absence of *stoS* or *sreKRS* did not attenuate virulence. To better understand the intrinsic functions of StoS and SreKRS, quantitative proteomics isobaric tags for relative and absolute quantitation (iTRAQ) was employed. Consistent with *stoS* and *sreK* mutants exhibiting a similar phenotype, the signalling circuits of StoS and SreKRS overlapped. Carbohydrate metabolism proteins and chemotaxis proteins, which could be responsible for EPS and swarming regulation, respectively, were reprogrammed in *stoS* and *sreK* mutants. Moreover, StoS and SreKRS demonstrated moderate expression of the major virulence factor, hypersensitive response and pathogenicity (Hrp) proteins through the HrpG-HrpX circuit. Most importantly, *Xoo* equipped with StoS and SreKRS outcompetes strains without StoS or SreKRS in co-infected rice and grows outside the host. Therefore, we propose that StoS and SreKRS adopt a novel strategy involving the moderation of Hrp protein expression and the promotion of EPS and motility to adapt to the environment.

The genus *Xanthomonas* comprises an important ubiquitous group of Gram-negative plant pathogenic bacteria belonging to the Gamma subdivision of Proteobacteria. Members of the genus *Xanthomonas* infect approximately 124 monocotyledonous and 268 dicotyledonous plants^1^. Bacterial blight resulting from *Xanthomonas oryzae* pv. *oryzae* (*Xoo*) infection is one of the most destructive rice diseases in tropical Asian countries, to which high-yielding rice cultivars are often highly susceptible. This bacteria has the potential to reduce rice yields by 20–30%, and the reduction can be as high as 50% in some areas^2^,^3^.

To survive and reproduce in the environment, bacteria require high fitness. For optimal fitness, bacteria must sense environmental changes; therefore, these microbes harbour an array of systems linking environmental stimuli to gene expression changes^4^. The two-component signal transduction system (TCS) is a predominant approach by which bacteria sense and adapt to changing intracellular and extracellular environments^5^. The prototypical TCS comprises a histidine kinase (HK) sensor protein, containing a cytoplasmic kinase core and an extrinsic membrane sensor domain, and a response regulator (RR) protein with a conserved receiver domain^6^. When a sensor domain recognizes a signal, the configuration of HK changes and the ATPase of the kinase core becomes activated to obtain a phosphate group from ATP^7^. The high-energy phosphate group of the histidine residue in HK is subsequently transferred to the aspartate residue of its cognate RR^7^. The activated RR regulates the downstream gene expression of its regulatory cascade as a transcription regulator or directly interacts with the target protein, resulting in an appropriate response^8^. To cope with various hostile stresses, *Xanthomonas* is equipped with a large number of TCSs^9^. In most bacterial genomes, dozens to hundreds of genes encode TCS proteins. In 2008, comparative genomic analyses of TCS genes in *Xanthomonas* were reviewed. For the six *Xanthomonas* strains investigated, the total number of nucleotides from TCS genes ranged from approximately 2.38% to 3.24% of the entire chromosome^10^.

Numerous TCS genes are present in *Xanthomonas*, but only some genes have been investigated. The most characterized TCS is RpfC/RpfG, which has been implicated in the perception and signal transduction of the quorum sensing diffusible signal factor^11^,^12^. Depending on the changes in the central pool of cyclic-di-GMP, a widely conserved novel second messenger implicated in the regulation of a wide range of bacterial functions, RpfC/RpfG regulates the biosynthesis of extracellular polysaccharide (EPS), extracellular cellulase, and eventually the virulence of *Xanthomonas*^13^. The OmpR-type RR, HrpG, the key regulator of hypersensitive responses and pathogenicity (*hrp*) genes, is an important TCS in *Xanthomonas*. HrpG, together with the transcriptional activator HrpX, regulates all 24 type III secretion system (T3SS) genes, the 23 T3SS effector (T3SSe) genes, and the 29 type II secretion system substrate genes^14^. Mutant studies have revealed that HrpG and HrpX from *Xanthomonas campestris* pv. *vesicatoria* are essential for pathogenicity^15^,^16^. Other TCSs, such as ColS/ColR and PdeK/PdeR, have also been reported to control the virulence factors of *Xanthomonas*^17^,^18^,^19^. However, the functions of many remaining TCSs in *Xanthomonas* remain elusive.

The present study started with a global investigation of EPS and swarming regulation applied to all HK genes in *Xoo* PXO99^A^. This investigation revealed that two TCSs, StoS (stress tolerance-related oxygen sensor) and SreKRS (salt response kinase, regulator, and sensor)^19^,^20^ regulate EPS synthesis and swarming, but not virulence in rice. To determine the intrinsic mechanisms and functions of StoS and SreKRS regulation, a quantitative proteomics analysis was performed. The global protein expression pattern revealed that StoS and SreKRS act as coordinators of virulence factors and confer high fitness to *Xoo* in rice and *in vitro*.

## Results and Discussion

### Systematic functional screening of two-component signal transduction systems

EPS blocks the water flow in rice xylem vessels and provides an advantage to *Xoo* against environmental stresses^1^. Swarming, bacterial group motility driven by flagellum, is considered a means for invading more territory in the natural habitats of bacteria and serves as an important virulence determinant in some species^21^. Here, we employed a genome-wide functional screen of TCS genes in *Xoo* PXO99^A^ based on EPS production and swarming ability. All 42 unique HK genes predicted using the P2CS database^9^ were knocked out in-frame through homologous double-crossover events using the suicide vector pK18mobsacB. All but three genes, *PXO_02837*, *PXO_03032*, and *PXO_*03565, among the 42 unique HK gene mutants were obtained.

**Figure 1:**
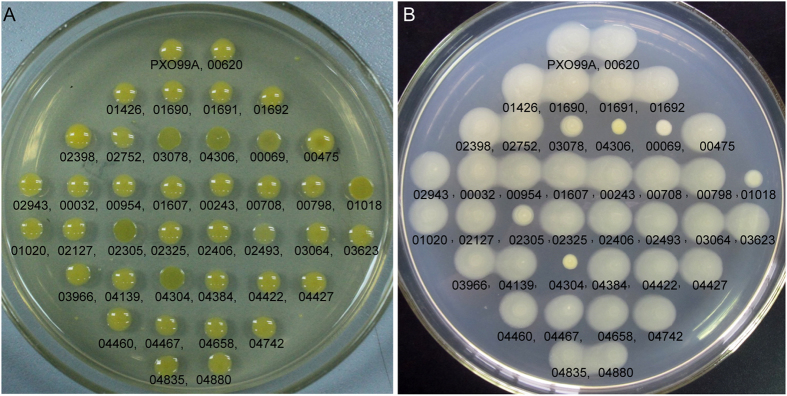
The EPS and swarming assays for all HK gene mutants. (**A**) In the EPS production test, a lustreless and applanate colony suggests low EPS production. (**B**) In the swarming assay, the colony size on the 18-cm diameter Petri dish indicates the swarming ability of *Xoo* strains. The numbers shown beneath the colonies are abbreviations for the mutants, e.g., 00620 indicates the mutant *PXO*Δ*00620*, in which *PXO_00620* was deleted.

An investigation into EPS production and swarming ability was performed after systematic gene deletion. Figure 1 shows that the deletion of *PXO_00069*, *PXO_01018*, *PXO_02305*, *PXO_03078*, *PXO_04304*, and *PXO_04306* in PXO99^A^ significantly attenuated EPS synthesis and swarming ability. The deletion of *PXO_00069* (*rpfC*) in PXO99^A^ attenuated EPS synthesis and swarming ability, consistent with previous reports^22^,^23^. However, the discovery that PXO_01018 (PdeK) and PXO_02305 (ColS) contributed to EPS synthesis and swarming improved our knowledge of PdeK/PdeR and ColS/ColR, which have been implicated in the regulation of several pathogenic factors but not EPS synthesis or swarming^17^,^18^,^24^. The other three EPS- and swarming-related HK genes, *PXO_03078*, *PXO_04304*, and *PXO_04306*, were selected for further study.

### StoS and SreKRS positively regulate EPS synthesis and swarming

PXO_03078, a hybrid HK, contains an HK kinase core, a RR receiver domain and two Per–ARNT–Sim sensor domains^25^. The orthologue of this gene in *Xoo* MAFF311018 StoS, which shares 100% coverage and 99% identity with PXO_03078, has recently been reported to be involved in stress tolerance^19^. Here, *PXO_03078* is also referred to as *stoS* for consistency. Moreover, two hypothetical genes, *PXO_03076* and *PXO_03077*, located upstream of *stoS* were predicted to form an operon with *stoS* using MicrobesOnline Operon Predictions (Supplementary Fig. S1)^26^. To validate the results of the bioinformatics analysis, reverse transcription polymerase chain reaction was performed on the predicted operons. Consistent with the prediction, *stoS*, *PXO_03077*, and *PXO_03076* are co-transcribed (Supplementary Fig. S1).

PXO_04304 is a typical HK with a Per–ARNT–Sim domain between the transmembrane helix and the HisKA domain, whereas PXO_04306 is a hybrid HK containing two receiver domains and a HisKA domain. Between these two proteins resides the RR PXO_04305. The orthologues of PXO_04304, PXO_04305 and PXO_04306 in *Xanthomonas campestris* pv. *campestris* 8004, which share 100% coverage and ≥85% identities, have been reported to fine tune the expression kinetics of 2-amino-4-hydroxy-6-hydroxymethyldihydropteridine pyrophosphokinase (*hppK*), another member of the *sreKRS* operon responsible for folate synthesis, via a positive feedback loop during stress responses^20^. To maintain consistency with their orthologues, *XC_0728*, *XC_0729*, and *XC_0730*, we refer to *PXO_04304*, *PXO_04305*, and *PXO_04306* as salt response kinase (*sreK*), salt response regulator (*sreR*), and salt response sensor (*sreS*), respectively.

**Figure 2:**
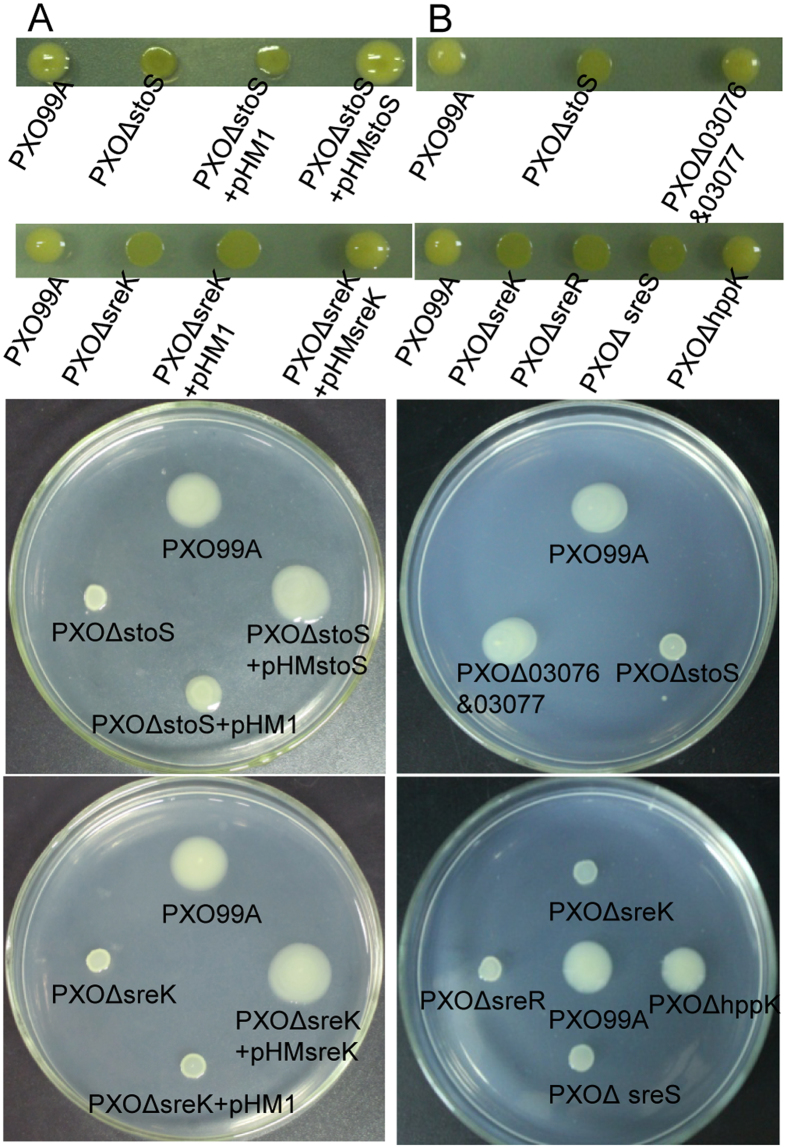
StoS and SreKRS positively regulate EPS synthesis and swarming. (**A**) Gene complementation analysis of *stoS* and *sreK*. The target genes were cloned into pHM1 and expressed in targeted mutants under the control of their native promoters and terminators. Mutants rescued with the deleted gene were applied to assay EPS production and swarming. (**B**) Other genes in the *stoS* and *sreKRS* operons were knocked out in-frame, and EPS production and swarming tests were performed on the mutants.

We firstly investigated the effects of *stoS* and *sreKRS* on bacterial growth. The growth curves of *PXO*Δ*stoS* and *PXO*Δ*sreK* were measured. No significant difference in growth between each mutant and the wild-type strain PXO99^A^ was observed when cultured in NB medium (Supplementary Fig. S2A). This result eliminated the possibility that the decline in EPS synthesis and swarming were caused by differences in their growth. A complementation experiment using *stoS* and *sreK* was performed to confirm that the phenotypic change resulted from the gene deletion. To restore the deleted genes under the control of their native promoters and terminators, the full ORFs of *stoS* or *sreK* and the sequences 500 bp upstream and 300 bp downstream of these operons were ligated via overlapping PCR (Supplementary Fig. S3) and cloned into the broad-host-range cloning vector pHM1, generating pHMstoS and pHMsreK, respectively. Figure 2A shows that *PXO*Δ*stoS* carrying pHMstoS exhibited EPS production and swarming similar to the wild-type strain PXO99^A^. Similarly, the EPS synthesis and swarming of *PXO*Δ*sreK* were restored through pHMsreK.

Other genes in the *stoS* and *sreKRS* operons were deleted to investigate genetic linkage within the operons. When *PXO_03077* and *PXO_03076* in the *stoS* operon were deleted, no discernible phenotypic changes were observed in the corresponding mutants compared with the wild-type strain PXO99^A^ (Fig. 2B). This finding indicates that *PXO_03077* and *PXO_03076* in the *stoS* operon are not involved in the signal transduction responsible for EPS synthesis and swarming. The deletion of *sreR* or *sreS* resulted in a dramatic decline in EPS production and swarming ability similar to *sreK* deletion mutants (Fig. 2B). The gene *hppK* (*PXO_04307*) in the *sreKRS* operon is another vital member of the signalling cascade of SreKRS in response to salt stress^20^. The deletion of *hppK* did not affect EPS production or swarming ability, indicating that HppK is independent of the regulation of EPS synthesis and swarming, and that this signal transduction circuit differed from the regulatory mechanism involving folate synthesis previously reported^20^. Taken together, these results demonstrated that StoS and SreKRS positively regulate EPS synthesis and swarming.

### Absence of StoS or SreKRS does not weaken the virulence of *Xoo*

**Figure 3:**
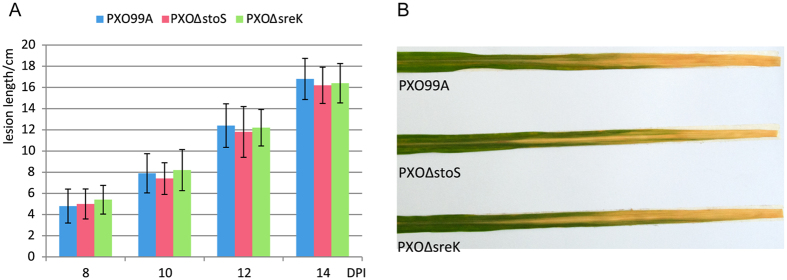
The absence of StoS or SreKRS does not weaken the virulence of *Xoo*. (**A**) Lesion lengths on rice leaves measured at 8, 10, 12 and 14 days after inoculation with *Xoo* strains PXO99^A^, *PXO*Δ*stoS*, and *PXO*Δ*sreK*. (**B**) Pictures of representative rice leaves were obtained at 14 days past inoculation.

StoS and SreKRS positively regulate EPS and swarming, which are important for the successful invasion of *Xanthomonas* into hosts. Furthermore, StoS and SreKRS have been previously implicated in hypoxia and high salt stress^19^,^20^, respectively. The involvement of StoS and SreKRS as stress resistance switches was further confirmed using hydrogen peroxide and osmotic pressure protection tests in the present study (Supplementary Fig. S4). To explore the final effect of the decline in the EPS production, swarming and stress tolerance in *stoS* and *sreK* mutants, a virulence assay was performed to identify pathogenicity changes. Surprisingly, when *PXO*Δ*stoS*, *PXO*Δ*sreK*, and wild-type PXO99^A^ were inoculated into the leaves of susceptive cultivar MH63 using the scissor-clip method, no obvious differences in the lesion length resulting from the mutants and wild-type strain were observed (Fig. 3A).

The plant pathogens environment is hostile and constantly changing, and TCS is a predominant means through which pathogens sense and adapt to the environment. Virulence is the most important characteristic for pathogens survival in host plants, the major natural habitats of plant pathogens. In the present study, two TCSs, StoS and SreKRS, were revealed to regulate several prominent virulence determinants but not directly attenuate virulence.

### General characterization of the iTRAQ analysis

**Figure 4:**
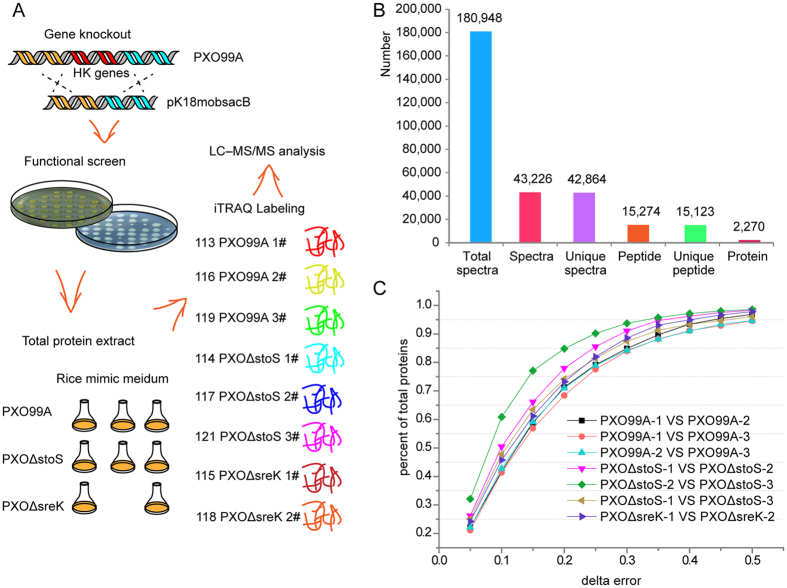
General characterization of the iTRAQ analysis. (**A**) Sample preparation process for the iTRAQ analysis. *PXO*Δ*stoS* and *PXO*Δ*sreK* are selected for quantitative proteomic iTRAQ analysis. Total protein was extracted from the strains cultured in XOM2, followed by trypsin digestion and labelling with 8-plex iTRAQ reagents. (**B**) Basic statistical information for iTRAQ. The data were acquired by LC-MS/MS and analysed using Mascot 2.3.02 against the genome of PXO99^A^ from NCBI (NC_010717.1). (**C**) Analysis of the reproducibility between the iTRAQ biological replicates of each treatment. The delta error means the variation between the fold change and 1, and the fold change is calculated between every two biological replicates. The vertical axis is the ratio of the number of proteins to the delta error in the total number of proteins quantified by comparisons between two biological replicates.

To further uncover the function of StoS and SreKRS, we identified differentially expressed proteins between PXO99^A^ and *PXO*Δ*stoS*/*PXO*Δ*sreK* using the proteomics approach iTRAQ. PXO99^A^, *PXO*Δ*stoS*, and *PXO*Δ*sreK* were cultured in the *hrp* genes induced medium XOM2. Total proteins were isolated when *Xoo* was grown to the middle of the logarithmic phase (OD_600_ = 0.9, Supplementary Fig. S2B). *PXO*Δ*stoS* and PXO99^A^ were analysed in three biological replicates, and *PXO*Δ*sreK* was analysed in two biological replicates. The proteins (100 μg) from each sample were digested with trypsin, labelled with 8-plex iTRAQ reagents, and subsequently mixed for LC-MS/MS analysis (Fig. 4A). A total of 180,948 mass spectra were generated from the eight samples, and 42,864 unique spectra were matched to specific peptides (15,123). Upon searching the genome of PXO99^A^, 2,270 proteins were identified using the 15,123 unique peptides (Fig. 4B). A cut-off point at ±50% variation (±0.50) generally yields 88% quantification coverage based on an analysis of the biological replicates^27^. Figure 4C shows that the coverage yielded at the 50% cut-off point in the iTRAQ data varied from 94.6% to 98.3%, indicating better reproducibility. Given that iTRAQ quantification might systematically underestimate ratios, a protein with ≥1.5-fold difference and *P* ≤ 0.05 is considered differentially expressed^28^,^29^. Compared with PXO99^A^, 46 proteins showed decreased levels and 36 proteins showed increased levels in *PXO*Δ*stoS* (Supplementary Tables S2 and S3). In *PXO*Δ*sreK*, 46 and 44 proteins showed decreased and increased levels, respectively (Supplementary Tables S2 and S3).

### StoS and SreKRS signalling circuits overlap

**Figure 5:**
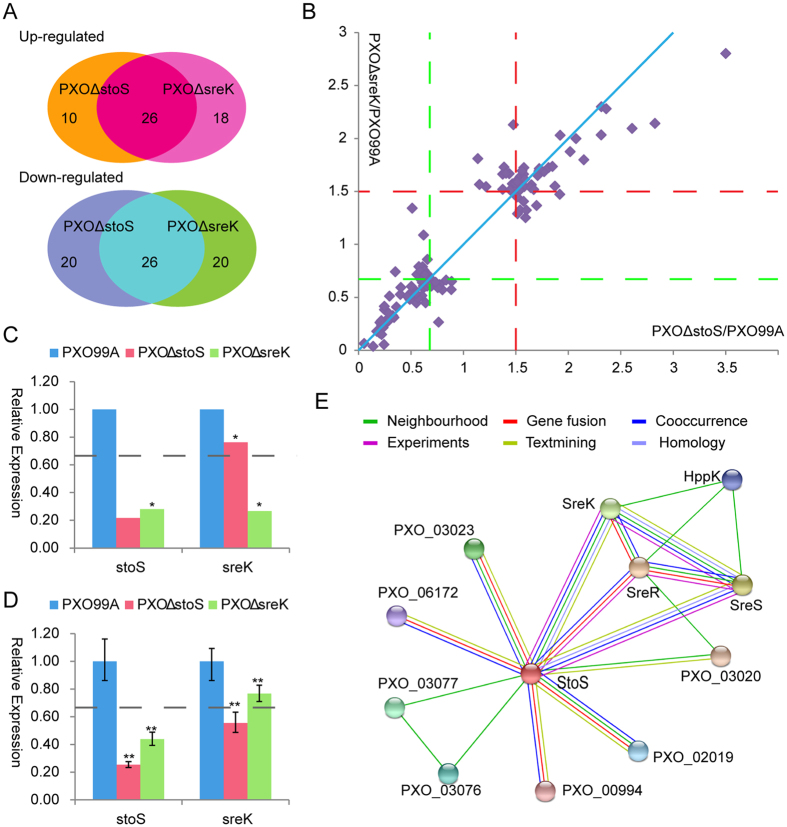
StoS and SreKRS overlapping signalling circuits. (**A**) Venn diagram showing that more than half of the differential expressed proteins in *PXO*Δ*stoS* and *PXO*Δ*sreK* overlap. (**B**) The differentially expressed proteins in *PXO*Δ*stoS* and *PXO*Δ*sreK* compared with the wild-type strain PXO99^A^ are shown together in a scatter diagram. (**C**) Relative expression levels of StoS and SreK in *PXO*Δ*stoS* and *PXO*Δ*sreK* compared with the wild-type strain PXO99^A^ identified by iTRAQ. (**D**) mRNA abundance of *stoS* and *sreK* in *PXO*Δ*stoS* and *PXO*Δ*sreK* compared with the wild-type strain PXO99^A^. The dotted lines indicate critical 1.5-fold up- or down-regulation. *denotes a statistical difference in which P ≤ 0.05, and **denotes P ≤ 0.01. (**E**) The relationship between StoS and SreKRS. Results of using StoS and SreKRS as queries in the STRING database. Lines in different colours represent special relationships between two proteins. PXO_03023, methyltransferase; PXO_06172, response regulator; PXO_03077 /PXO_03076, hypothetical protein; PXO_03020, transcriptional regulator NtrC family; PXO_02019, putative signal protein with GGDEF domain; PXO_00994, response regulator.

Figure 5A shows that more than half of the differentially expressed proteins (26 up-regulated proteins and 26 down-regulated proteins) in *PXO*Δ*stoS* were also regulated by SreK. Moreover, these proteins were up- or down-regulated to similar degrees in the *PXO*Δ*stoS* and *PXO*Δ*sreK* mutants (Fig. 5B), suggesting interplay between the signal transduction and regulatory circuits associated with StoS and SreKRS in PXO99^A^. Notably, StoS was significantly down-regulated by 3.6-fold in the mutant *PXO*Δ*sreK* compared with PXO99^A^ (Supplementary Table S2, Fig. 5C). SreK expression was also weakened by 1.3- and 3.8-fold in *PXO*Δ*stoS* and *PXO*Δ*sreK*, respectively, detected by the remaining SreK peptide in iTRAQ. The transcription of the remaining segments of *stoS* and *sreK* was measured in *PXO*Δ*stoS*, *PXO*Δ*sreK*, and the wild-type strain PXO99^A^. Figure 5D shows that the transcription levels of *stoS* and *sreK* were significantly down-regulated in both mutants compared with the levels of these genes in PXO99^A^. This result suggests that StoS expression is positively regulated by itself and by SreKRS, and that the *sreKRS* operon is also activated by StoS and SreKRS.

To further explore the relationship between StoS and SreKRS, these proteins were used as queries in the STRING database, which assembles, evaluates, and disseminates information concerning protein-protein associations^30^. As shown in Fig. 5E, the homologous genes of *stoS* and *sreK* are neighbours in some bacterial genomes, such as *baeS* and *baeR* in *Escherichia coli* K12_MG1655. Putative homologues of StoS and SreK, such as ArcB and CheY in *E. coli* EC4115, were found to interact. Furthermore, *stoS* and *sreKRS* co-occur in many genomes, particularly in *Xanthomonas* group genomes. Again, this information indicates that StoS and SreKRS are closely connected and their circuits overlap. The overlapped signalling circuits of StoS and SreKRS elucidate similar phenotypes in *stoS* and *sreK* mutants.

Genome-wide microarray analysis of all TCS mutants in *Escherichia coli* suggests that signal circuits of numerous TCSs overlap^31^,^32^. Cross-regulation facilitates the elicitation of some of the same responses through distinct pathways^31^,^32^. The domain architectures of StoS and SreKRS are markedly different from each other, suggesting that StoS and SreKRS might be activated through distinct signals. We proposed that StoS and SreKRS could sense different stimuli to express similar virulence factors, resulting from overlapping pathways. This pathogen takes advantage of the connection between diverse functional modules for survival in hostile environments.

### The EPS and swarming regulatory circuit of StoS and SreKRS

**Figure 6:**
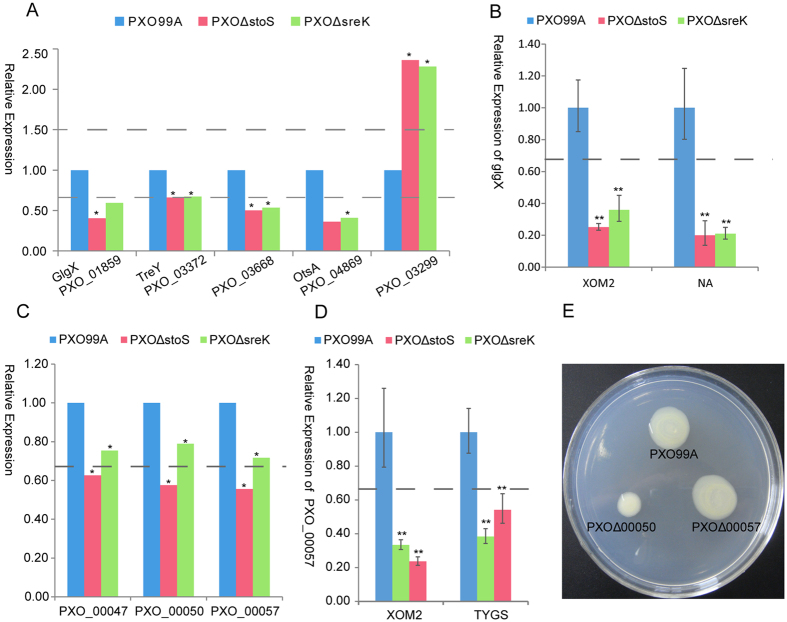
EPS synthesis- and swarming-regulated proteins were down-regulated in *PXO*Δ*stoS* and *PXO*Δ*sreK*. (**A**) Several carbohydrate metabolic pathway proteins (GlgX, glycogen debranching protein; TreY (PXO_03372), malto-oligosyltrehalose synthase; PXO_03668, alpha-amylase family protein; OtsA (PXO_04869), alpha, alpha-trehalose-phosphate synthase and PXO_03299, endoglucanase) were differentially expressed in *PXO*Δ*stoS* and *PXO*Δ*sreK* compared with the wild-type strain PXO99^A^, as identified by iTRAQ. (**B**) The expression difference in glycogen debranching protein GlgX was confirmed at the mRNA level. (**C**) Three chemotaxis proteins, PXO_00047, PXO_00050, and PXO_00057, were down-regulated in *PXO*Δ*stoS* and *PXO*Δ*sreK* compared with the wild-type strain PXO99^A^, as identified by iTRAQ. (**D**) The expression difference for *PXO_00057* was confirmed by qRT-PCR. The dotted lines indicate a critical 1.5-fold down-regulation. *denotes a statistical difference in which P ≤ 0.05, and **denotes P ≤ 0.01. (**E**) *PXO_00050* and *PXO_00057* were deleted, generating *PXO*Δ*00050* and *PXO*Δ*00057*. The swarming ability of these mutants was measured by colony size in semi-solid medium.

EPS produced by *Xanthomonas* consists of D-glucosyl, D-mannosyl, and D-glucuronyl acid residues at a molar ratio of 2:2:1 and variable proportions of O-acetyl and pyruvyl residues^33^. This EPS is assembled stepwise from UDP-glucose, GDP-mannose, and UDP-glucuronic acid, which are also important substrates of many carbohydrate metabolic pathways, such as starch and sucrose metabolism and galactose metabolism (http://www.genome.jp/kegg/kegg2.html). The synthesis of EPS could also be affected by other carbohydrate metabolic pathways. The pathway enrichment analysis showed that the *Xoo* mutants might reprogram starch and sucrose metabolism through the regulation of four proteins in *PXO*Δ*stoS* and three proteins in *PXO*Δ*sreK* (*P* = 0.0033 and 0.0401, respectively) (Fig. 6A; Supplementary Tables S4 and S5). In addition to starch and sucrose metabolism enrichment, the galactose metabolism pathway was enriched by differentially expressed proteins in *PXO*Δ*stoS* (P = 0.0016) and *PXO*Δ*sreK* (P = 0.0341) (Supplementary Tables S4 and S5). The differential expression of the glycogen debranching protein GlgX (PXO_01859) in the starch and sucrose metabolism pathways was verified at the mRNA level by qRT-PCR (Fig. 6B). Because EPS production was tested on NA medium, the expression differences of *glgX* were further confirmed by measuring the mRNA abundance in bacteria incubated on NA medium. Regardless of culture in XOM2 or on NA medium, *glgX* expression differences between mutants and wild type were consistent with the results of the iTRAQ analysis (Fig. 6B). These results indicated that the carbohydrate metabolism in *PXO*Δ*stoS* and *PXO*Δ*sreK* was reprogrammed, which might be involved in the attenuation of EPS synthesis. In *Xanthomonas*, gum gene cluster proteins were recognized to be response for the biosynthesis of EPS and were extremely important in EPS production^34^. The gum gene cluster comprises a tandem array of 12 ORFs, from gumB to gumM. All 12 proteins were identified by iTRAQ (data not shown), and two of these proteins, GumB and GumE, were up-regulated in *PXO*Δ*stoS* and *PXO*Δ*sreK* compared with the wild-type strain PXO99^A^ (Supplementary Table S3). GumB and GumE might be required for the polymerization of EPS subunits or the export of the polymer^33^. The overexpression of GumB and GumE in *PXO*Δ*stoS* and *PXO*Δ*sreK* might be a compensatory measure for reduced EPS production.

Swarming, an important virulence determinant, was sharply attenuated in *PXO*Δ*stoS* and *PXO*Δ*sreK*. The iTRAQ data showed that the expression levels of the three chemotaxis genes, *PXO_00047*, *PXO_00050*, and *PXO_00057*, declined more than 1.5-fold in *PXO*Δ*stoS* and 1.2-fold in *PXO*Δ*sreK* compared with the wild-type strain PXO99^A^ (Fig. 6C). qRT-PCR was employed to detect the mRNA abundance in the *Xoo* sample cultured in XOM2 and on TYGS medium, consistent with the culture conditions used for the swarming ability test. Figure 6D shows that *PXO_00057* expression at the mRNA level decreased more than 1.5-fold in *PXO*Δ*stoS* and *PXO*Δ*sreK*. To further explore the role of chemotaxis proteins in swarming, *PXO_00050* and *PXO_00057* were deleted in-frame. Although the absence of chemotaxis-specific methylesterase PXO_00057 does not affect swarming, the chemotaxis signal transduction protein PXO_00050 contributes to the motility of *Xanthomonas* (Fig. 6E). These results indicated that chemotaxis could be one of the circuits to regulate the swarming of *Xoo*.

### StoS and SreKRS fine-tune *hrp* and T3SSe genes expression

**Figure 7:**
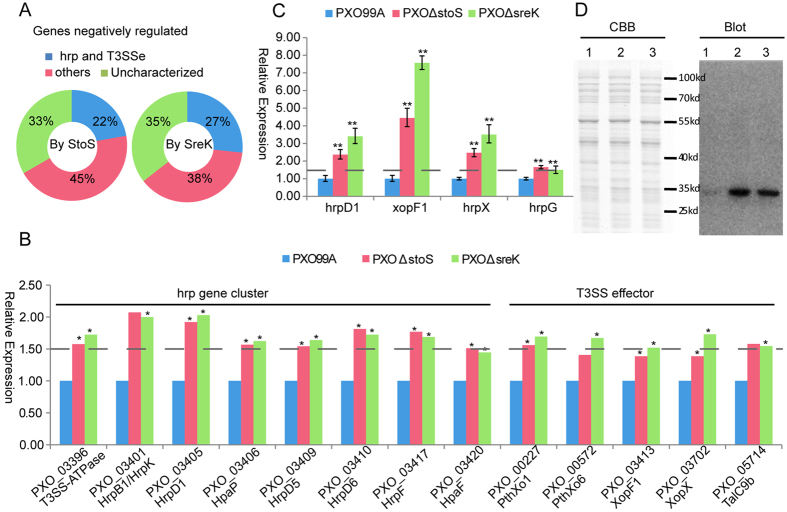
StoS and SreKRS negatively regulate *hrp* and T3SSe gene expression through the HrpG-HrpX circuit. (**A**) The proportion of *hrp* and T3SSe genes in the list of up-regulated proteins of *PXO*Δ*stoS* and *PXO*Δ*sreK* compared with wild-type PXO99^A^. (**B**) iTRAQ data shows that Hrp proteins and T3SSe were negatively regulated by StoS and SreKRS. (**C**) The relative expression levels of type III secretion apparatus protein HrpD1 and XopF1 effectors were measured by qRT-PCR. The relative expression of *hrpX* and *hrpG* in *PXO*Δ*stoS* and *PXO*Δ*sreK* compared with the wild-type PXO99^A^ was also determined by qRT-PCR. (**D**) HrpG quantitative analysis at the post-transcriptional level by western blot analysis. The results of Coomassie brilliant blue (CBB) staining show similar quantities in different lanes. Western blot and CBB staining were under the same experimental conditions for protein sample preparation and SDS-PAGE. Lane 1, *hrpGHis/PXO*Δ*hrpG*. Lane 2, *hrpGHis/PXO*Δ*hrpG&stoS* Lane 3, *hrpGHis/PXO*Δ*hrpG&sreK.* The dotted lines indicate the critical 1.5-fold up-regulation. *denotes a statistical difference in which P ≤ 0.05, and **denotes P ≤ 0.01.

The *hrp* genes are extremely important for pathogenicity. Hrp proteins include T3SS regulon regulatory proteins, pilus components, and proteins functioning at different stages of injectisome assembly^35^. These *hrp* genes were clustered in a 28-kb pathogenicity island in PXO99^A^. T3SSe can be injected into the host cell cytosol to manipulate plant cellular processes, such as basal defences, to benefit the pathogen^1^. Typically, T3SSe encoding genes are scattered throughout the chromosome. Among the up-regulated proteins, Hrp proteins together with the T3SSe accounted for 22% in *PXO*Δ*stoS* and 27% in *PXO*Δ*sreK* (Fig. 7A). As shown in Fig. 7B, eight *hrp* genes or T3SSe encoding genes in *PXO*Δ*stoS* and 12 *hrp* genes or T3SSe encoding genes in *PXO*Δ*sreK* were up-regulated more than 1.5-fold compared with PXO99^A^. Seven identical genes were both up-regulated in *PXO*Δ*stoS* and *PXO*Δ*sreK*. A total of 13 up-regulated unique *hrp* genes or T3SSe encoding genes were identified in the two mutants. Eight of the 13 genes were clustered in a *hrp* pathogenicity island. The other five genes encoding T3SSe were scattered throughout the chromosome (Fig. 7B). The differential expression of the type III secretion apparatus protein HrpD1 and the type III effector XopF1 in mutant and wild-type bacteria was confirmed by qRT-PCR (Fig. 7C). These results suggest that StoS and SreKRS modulate the expression of *hrp* and T3SSe genes.

To minimize metabolic consumption, *hrp* and T3SSe genes are not constitutively expressed but rather become activated when *Xanthomonas* enters the host or is cultured in certain minimal media^36^. All genes, except *hrpA* in *Xanthomonas*, are under the direct transcriptional control of HrpX (PXO_01953), a transcription regulator that is transcriptionally activated by the global response regulator, HrpG^35^. Therefore, the expression levels of *hrpX* and *hrpG* in *PXO*Δ*stoS* and *PXO*Δ*sreK* were detected using qRT-PCR. The experimental results verified the hypothesis that the mRNA abundance of *hrpX* and *hrpG* was significantly increased in *PXO*Δ*stoS* and *PXO*Δ*sreK* compared with that in PXO99^A^, indicating that the negative regulation of *hrp* and T3SSe genes occurs through the HrpG-HrpX circuit.

Because none of the SreKRS and StoS members possess the helix-turn-helix domain, the essential domain of a typical transcriptional regulator, StoS and SreKRS might not directly regulate *hrpG* at the transcriptional level. HrpG plays global roles in the signalling network of *Xanthomonas*. Interestingly, *hrpG* exists in the list of HrpG positively regulated gene^14^, suggesting that HrpG potentially possesses self-regulation activity. Therefore, we hypothesized that the differential expression of *hrp* and T3SSe encoding genes resulting from StoS and SreKRS was implemented by HrpG self-regulation. The quantitative analysis of HrpG protein at the post-transcriptional level was performed in mutants and wild type. HrpG fused with a 6*His tag was under the control of the constitutive lac promoter in a broad-host-range cloning vectors pBBR1MCS4_START^37^. HrpGHis was subsequently trans-expressed in the *hrpG* mutant, the *hrpG* and *stoS* double mutant, and the *hrpG* and *sreK* double mutant. The HrpGHis quantitation on different genetic backgrounds was detected by western blot with monoclonal antibody against 6*His tag. As shown in Fig. 7D, HrpG is sharply up-regulated because of the absence of *stoS* or *sreK*. This result shows that StoS and SreKRS regulate HrpG at the post-transcriptional level. Taken together, StoS and SreKRS decrease HrpG at the post-transcriptional level, subsequently reduce *hrpG* at the transcriptional level by feedback regulation, and moderate the expression of all *hrp* and T3SSe genes.

### StoS and SreKRS contribute to the full fitness of *Xoo in vivo* and *in vitro*

EPS produced by *Xanthomonas* is highly hydrated and anionic consistency, and protects bacteria from environmental stresses^1^. Swarming is a powerful means of invading more territory in the natural habitat and accelerating the biomass production of bacteria^38^. Hrp proteins, major virulence factors of *Xanthomonas*, are extremely important for pathogenicity. However, virulence traits carry a significant cost to the pathogen^39^, and redundant virulence factors induce enormous metabolic consumption and are disadvantageous to bacterial fitness^40^,^41^,^42^. StoS and SreKRS finely orchestrated these virulence factors and responded to stress tolerance as previously reported^19^,^20^. Moreover, the growth of the *stoS* and the *sreK* mutants are significantly inferior to that of the wild-type strain growth in XOM2 (Supplementary Fig. S2). Based on these facts, we hypothesized that StoS and SreKRS are essential for *Xoo* fitness in rice, although StoS and SreKRS do not directly control virulence.

**Figure 8:**
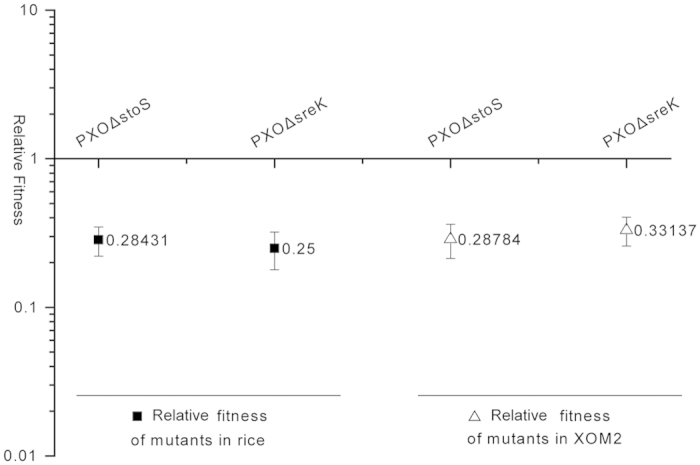
Relative fitness of *stoS* and *sreK* mutants in rice and XOM2 competed with wild-type strain PXO99^A^. PXO99^A^ and mutants were mixed at a ratio of 1:1 and inoculated into rice leaves and XOM2 medium. The ratio between mutant and PXO99^A^ colony forming units was measured at 10 days after inoculating to rice and 60 hours after inoculating to XOM2. This ratio was defined as the relative fitness of *PXO*Δ*stoS* and *PXO*Δ*sreK*

To verify this hypothesis, we measured the relative fitness of *PXO*Δ*stoS* and *PXO*Δ*sreK* in rice and in XOM2 using a competition assay^43^. The mutants and wild-type strains were mixed at a ratio of 1:1 and inoculated into rice leaves and XOM2. The relative fitness indices of the mutants were calculated by dividing mutants colonies number by wild-types colonies number. As shown in Fig. 8, the relative fitness index of *PXO*Δ*stoS* was 0.28 when co-infected with PXO99^A^ in rice. Similarly, the absence of *sreK* reduced the fitness in rice to 0.25. When co-cultured in XOM2 with the wild-type strain, *stoS* and *sreK* mutants were outcompeted, with relative fitness indices of 0.29 and 0.33, respectively. This finding shows that StoS and SreKRS confer *Xoo* fitness for infection rice and growth independent of host plants.

Genotypes with higher fitness will tend to produce more offspring and thereby increase in frequency over time compared with less-fit competitors^43^. Although *stoS* and *sreK* mutants did not show obvious virulence weakness under laboratory condition, as which is constant and benefit to *Xoo* infection, *Xoo* strains without StoS or SreKRS might be eliminated in hostile natural habitats evolved with competitors and environmental stresses.

## Conclusion

**Figure 9:**
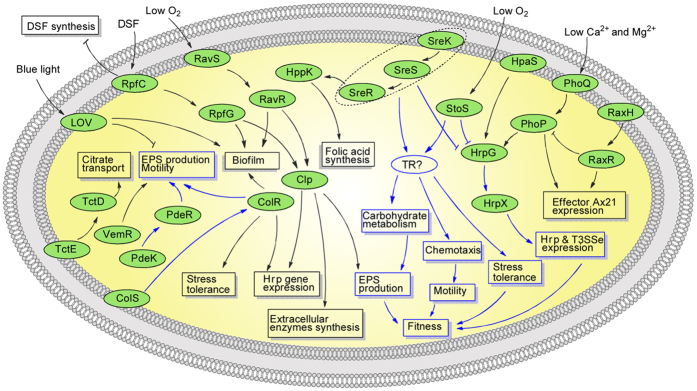
TCS network in *Xanthomonas*. All of the functionally characterized TCSs in *Xanthomonas* are updated in this figure. The signalling circuits depicted in blue were identified in the present study, and other circuits were summarized from previous studies (RpfC/G^13^, HpaS/HrpG^53^, RavS/R^54^, ColS/R^18^,^24^, PhoQ/P^55^, RaxH/R^56^,^57^, LOV^58^, PdeK/R^17^, TctE/D^59^, and VemR^48^). The characterized SreKRS circuit is represented by dotted lines, because which component of the SreKRS is in charge of the signal output is still unknown. The differentially expressed genes might be not directly regulated by StoS or SreKRS but are regulated by other unknown transcriptional regulator (TR?). Lines with arrowheads represent signal transduction or positive regulation. “T” lines indicate negative regulation.

The TCS is a prevalent strategy for coupling environmental stimuli to adaptive responses in bacteria^6^. In most bacterial genomes, dozens to hundreds of genes encode TCS proteins. However, most of these TCSs remain functionally unknown. In the present study, we revealed that StoS and SreKRS confer fitness to *Xoo* by contributing to EPS production, motility and stress tolerance, and moderating the expression of Hrp proteins. This mechanism is supposed to be a novel strategy to adapt to changing environments. These findings show the roles of StoS and SreKRS in the network of TCS-mediated environmental adaptation. StoS, SreKRS, and the previously characterized TCSs form an intricate signalling network, providing *Xanthomonas* with an economic and effective gene expression regulation mechanism to adapt to changing intra- and extracellular environments. Figure 9 shows an updated model of the TCS network, in which the signalling circuits uncovered in the present study are highlighted in blue. These results improved the current knowledge of the TCS network in *Xanthomonas*. However, the exact details of StoS and SreKRS regulatory mechanisms remain unknown. The negative regulation of Hrp proteins has drawn much attention, and the regulatory mechanism is worthy of further exploration.

## Methods

### Strains, plasmids, and culture conditions

*Xoo* PXO99^A^, 5-azacytidine-resistant derivative of Philippine race 6 PXO99, was kindly presented by Dr. Ya-Wen He^44^. The plasmid used for gene knock-out pK18mobsacB was offered by Dr. Jin He. The broad-host-range cosmid used for gene rescue pHM1 was a gift from Dr. Gong-You Chen in Shanghai Jiao Tong University^45^. Mutants obtained in this study are listed in Supplementary Table S1. *Xanthomonas* strains were cultivated at 28 °C in NB medium (beef extract, 3 g/L; yeast extract, 1 g/L; polypeptone, 5 g/L; and sucrose, 10 g/L) or on solid NB medium added with 1.5% agar (NA) except for special circumstances. When required, growth media were supplemented with antibiotics at the following final concentrations: kanamycin, 25 μg/mL; spectinomycin, 50 μg/mL; ampicillin, 100 μg/ml.

### Gene deletion in *Xoo*

All the HK genes in PXO99^A^ were designed to generate mutants via allelic homologous recombination. In brief, two flanking regions of the target gene were cloned to the suicide plasmid pK18mobsacB by three appropriate restriction sites^46^ (Supplementary Table S1). For the convenience of the clone and efficiency of recombination, the lengths of homologous DNA fragments were between 500 and 900 bp. The recombinant plasmid verified by sequencing was subsequently imported into the wild-type strain PXO99^A^ via electroporation to generate one cross-over mutant. This cross-over mutant was then cultured in NAS medium (NA containing 10% sucrose) to generate a second cross-over. Strains which are able to grow on NAS lost the plasmid because of the second cross-over event that either restored the wild-type situation or led to the gene deletion mutant, which could be sorted by PCR.

### Gene complementation

For complementation analysis, the ORF region of the target gene, up-stream 500 bp segment which was predicted as promoter, and down-stream 300 bp segment which was predicted as terminator were amplified and ligated by overlapping PCR (Supplementary Table S1). The target gene under the control of its native promoter and terminator was then cloned to a broad-host-range cosmid pHM1. The recombinant vector was electroporated into the mutant to analyze the complementation^17^.

### EPS production and swarming ability determination

An effective and visualized method was applied to analyze EPS production^47^. In brief, strains were cultured in NB medium and adjusted to the same concentration. EPS production was detected by the appearance of colonies arising in NA medium after 48 to 72 hours from 2 μL of culture drops. Low EPS production would lead to a lustreless and applanate colony. Swarming ability was measured by colony dimension arising in semi-solid medium TYGS (tryptone, 1 g/L; yeast extract, 0.5 g/L; glucose, 10 g/L; NaCl, 1 g/L; agar 0.6%) from 2 μL drops of the equivalent concentration cultures^48^,^49^. The experiments were repeated at least three times.

### Virulence assay

The susceptible rice breed MH63 was utilized to analyze the virulence of the *Xoo* strains. In brief, five rice plants were grown per circular pot (about 80 mm^2^) in a tray containing standing water for 4–5 weeks, while *Xoo* strains were cultured in NB medium for 24–48 hours to late logarithmic phase. All the strains were adjusted to OD_600_ = 1.0. Two youngest fully expanded leaves of the prepared plants were clipped about 2cm from the tip with scissors that have been immersed in a bacterial suspension immediately prior to each clipping. All the five plants in one pot were used for inoculation of each *Xoo* strain. The inoculated rice plants were grown in the green house under 28–35 °C with 75% relative humidity. Lesion lengths of the diseased leaf were measured at 14 days after inoculation^50^. The experiments were repeated at least three times.

### Protein preparation for isobaric tags for relative and absolute quantitation

*Xoo* strains were grown in XOM2^51^ (D-xylose, 0.18%; D, L-methionine, 670 μM; sodium L(+)-glutamate, 10 mM; KH_2_PO_4_, 14.7 mM; MnSO_4_, 40 μM; Fe(III)-EDTA, 240 μM; and 5 mM MgCl_2_) to the middle logarithmic phase (OD_600_ = 0.9). The harvested cultures were washed twice with PBS (137 mM NaCl; 2.7 mM KCl; 10 mM Na_2_HPO_4_; and 2 mM KH_2_PO_4_, pH 7.4). The pellets were subsequently resuspended in 10 mL of PBS containing a tablet of protease inhibitor cocktail (Roche Diagnostics, Mannheim, Germany) per 100 mL of culture, followed by cell disruption using a low temperature ultra-high pressure continuous cell disrupter. The disrupted cells were centrifuged at 12,000 rpm and 4 °C for 20 min to remove cell debris, and the supernatant containing soluble proteins was precipitated with precooling acetone (25 mL per 5 mL of supernatant) overnight at −20 °C. The precipitates were washed twice with cold acetone, and the proteins were dissolved in 7 M urea and 2 M thiourea. A reductive alkylation reaction was subsequently applied to each protein sample, followed by acetone precipitation and dissolution in 0.5 M tetraethylammonium bromide. Finally, the protein concentration was determined using the Bradford method.

### Isobaric peptide labeling and Strong Cation Exchange fractionation

The proteins (100 μg) from each sample were digested with trypsin and labelled with 8-plex isobaric tags for relative and absolute quantitation (iTRAQ) reagents according to the manufacturer’s protocol. All samples were labelled as follows: PXO99^A^1#_113, PXO99^A^2#_116, PXO99^A^3#_119, *PXO*Δ*stoS*1#_114, *PXO*Δ*stoS*2#_117, *PXO*Δ*stoS*3#_121, *PXO*Δ*sreK*1#_115, and *PXO*Δ*sreK*2#_118. All labelled samples were pooled and dissolved in 4 mL of buffer A (25 mM NaH_2_PO_4_ in 25% ACN, pH 2.7). Peptides were fractioned using a 4.6 mm × 250 mm UltremexSCX column on a Prominence LC-20AB HPLC system with a constant flow rate of 1 mL/min. A 22 min gradient consisting of 100% buffer A for 10 min, 5% to 35% buffer B (25 mM NaH_2_PO_4_, 1 M KCl in 25% ACN, pH 2.7) for 11 min, and 35% to 80% buffer B for 1 min was performed. The chromatogram was monitored under 214 nm. A total of 12 fractions were obtained from the Strong Cation Exchange Choematography separation, and each fraction was desalted and dried.

### LC–MS/MS

The LC–MS/MS package and system parameters were similar to those previously described^28^. Briefly, peptides from Strong Cation Exchange fractions were enriched and desalted using Symmetry C18 Trap column (5 μm, 180 μm × 20 mm), and separated via BEH130 C18 column performing on nanoACQuity (Waters, USA) liquid chromatography system. Data was acquired by Triple TOF 5600 (AB SCIEX, Concord, ON, USA) fitted with a Nanospray III source (AB SCIEX, USA) and a pulled quartz tip as the emitter (New Objectives, USA).

### Data analysis

Protein identification and quantification were carried out using Mascot 2.3.02 against the genome of PXO99^A^ from NCBI (RefSeq accession number NC_010717.1). Other search parameters were as follows: type of search, MS/MS ion search; enzyme, trypsin; fragment mass tolerance, ±0.05 Da; mass values, monoisotopic; variable modifications, Gln->pyro-Glu (N-term Q), oxidation (M), iTRAQ8plex (Y); peptide mass tolerance, ±0.1 Da; instrument type, default; max missed cleavages, 1; and fixed modifications, carbamidomethyl (C), iTRAQ8plex (N-term), and iTRAQ8plex (K). For protein identification and quantification, *P* ≤ 0.05 (with 95% confidence) was considered statistically significant. The mass spectrometry proteomics data have been deposited to the ProteomeXchange Consortium^52^ via the PRIDE partner repository with the dataset identifier PXD002625. The results obtained were exported as a Microsoft Excel file for further analysis.

Pathway enrichment analysis was performed to identify the significantly enriched pathways. Briefly, all the differential expressed proteins were mapped to Kyoto Encyclopedia of Genes and Genomes (KEGG) database, and the number of every mapped pathway term was acquired. Hypergeometric test was then employed to find the enriched pathways using all the proteins identified by iTRAQ as background data.

### Real-time quantitative reverse transcription PCR

To verify gene expression differences identified by iTRAQ, *Xoo* were cultured in XOM2 to the middle logarithmic phase (OD_600_ = 0.9) for RNA extraction. Consistent with the culture conditions used for the EPS production and swarming tests, *Xoo* strains were also cultured on NA (NB added with 1.5% agar) or TYGS semi-solid medium for approximately 60 hours, and the bacteria were collected from NA or TYGS for RNA extraction. RNA was extracted using the RNApure Bacteria Kit (Beijing CoWin Biotech, Beijing, China) according to the manufacturer’s instructions. RNA integrity was confirmed by electrophoresis on 0.8% agarose gels. The RNA purity and concentration were determined by NanoDrop 2000 (Thermo Fisher Scientific, Waltham, MA, USA). cDNA was synthesized from total RNA (2 μg) using a SuperRT cDNA Kit (Beijing CoWin Biotech, Beijing, China). The primers used for real-time quantitative reverse transcription PCR (qRT-PCR) are listed in Supplementary Table S1. The qRT-PCR reaction was performed using SYBR^®^ Select Master Mix (Applied Biosystems, Foster City, CA, USA) on a ViiA 7 PCR system (Applied Biosystems, Foster City, CA, USA), and 16s rRNA was used as an endogenous control for gene expression analysis by ΔΔC_T_. All genes were investigated with three technological repeats, and ANOVA for differences in C_T_ values (ΔC_T_) was performed to assess whether a statistical difference exists in gene expression level between the mutant and wild-type strains. The experiments were repeated at least three times.

### Quantitative analysis of HrpG protein by western blotting

The *hrpG* and *stoS/sreK* double mutants, *PXO*Δ*hrpG&stoS* and *PXO*Δ*hrpG&sreK*, were generated by knocking out *stoS* or *sreK* in the *hrpG* mutant *PXO*Δ*hrpG*. The *hrpG* ORF was amplified by the primers in Supplementary Table S1, and the sequence encoding the 6*His tag was added to the 5′ terminal of the reverse primer to fuse the 6*His tag to the C terminal of the HrpG protein. *hrpGHis* was subsequently cloned into pBBR1MCS4_START^37^, generating pBBRhrpG. pBBRhrpG was imported into *PXO*Δ*hrpG*, *PXO*Δ*hrpG&stoS* and *PXO*Δ*hrpG&sreK* to quantitatively analyse HrpGHis protein under different genetic backgrounds.

*Xoo* strains were cultured in XOM2 to the middle logarithmic phase (OD600 = 0.9) and harvested by centrifugation. The pellets were subsequently resuspended in PBS and disrupted using an ultrasonic processor. Supernatants containing soluble proteins were used for SDS-PAGE and western blot analyses. A monoclonal antibody against 6*His and the HRP-conjugated secondary antibody were commercially purchased from Proteintech (Wuhan, China). Western blot analysis was performed using a general method, and the proteins were detected by chemiluminescent detector system using Pierce ECL (Thermo Fisher Scientific, Waltham, MA, USA) as a HRP Substrate.

### Relative fitness assay

Wild-type and mutant *Xoo* strains were cultured in NB medium for approximately 24 hours to the middle logarithmic phase and adjusted to OD600 = 1.0. The adjusted wild-type and mutant *Xoo* strains were mixed at a ratio of 1:1. The mixed culture was subsequently inoculated into rice leaves and XOM2 medium as described above to assay the relative fitness of *Xoo* in rice and *in vitro*, respectively. For the *in vivo* assay, the diseased rice leaves were cut from the plants at 10 days after inoculation, sterilized in 70% ethanol for 30 seconds, rinsed three times in sterile water, and subsequently homogenized and plated on NA medium after serial dilution. Similarly, culture in XOM2 was serially diluted and plated on NA medium at 60 hours after inoculation. *Xoo* colonies on NA were counted at 72 hours after plating. The s*toS* and *sreK* mutant colonies were lustreless and applanate, whereas the colonies from the wild-type strain PXO99^A^ had lustre and were raised. The relative fitness of the mutant was defined as the ratio of mutant colonies and PXO99^A^ colonies. The experiments were repeated at least three times.

## Additional Information

**How to cite this article**: Zheng, D. *et al.* Two overlapping two-component systems in *Xanthomonas oryzae* pv. *oryzae* contribute to full fitness in rice by regulating virulence factors expression. *Sci. Rep.* **6**, 22768; doi: 10.1038/srep22768 (2016).

## Supplementary Material

Supplementary Information

Supplementary Tables
