# Supplementary material for: Two overlapping two-component systems in Xanthomonas oryzae pv. oryzae contribute to full fitness in rice by regulating virulence factors expression
Source: Sci Rep. 2016 Mar 9;6:22768. doi: 10.1038/srep22768 (PMC4783713; doi:10.1038/srep22768)
Supplement: Supplementary Information [file srep22768-s1.pdf]

Two overlapping two-component systems in *Xanthomonas oryzae* pv. *oryzae* contribute to full fitness in rice by regulating virulence factors expression

Dehong Zheng, Xiaoyan Yao, Meng Duan, Yufeng Luo, Biao Liu, Pengyuan Qi, Ming Sun, Lifang Ruan*

State Key Laboratory of Agricultural Microbiology, College of Life Science and Technology, Huazhong Agricultural University, Wuhan 430070, PR China

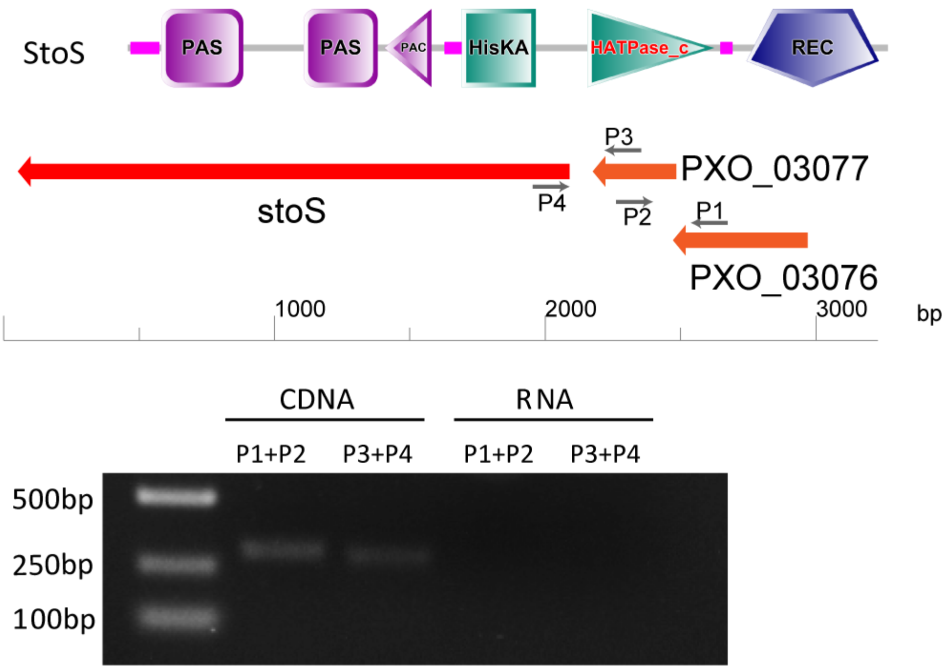

Figure S1. Bioinformatics analysis of StoS. Conserved domains of StoS were analyzed using SMART database. PAS: PAS domain, PAC: Motif C-terminal to PAS motifs, HisKA: His Kinase A (phosphoacceptor) domain, HATPase_c: Histidine kinase-like ATPases, REC: cheY-homologous receiver domain. Operon consisting of *stoS*, *PXO_03077* and *PXO_03076* were predicted by Microbesonline Operon Predictions (http://vimss.org/operons) and verified by RT-PCR. cDNA used for PCR template was reverse-transcribed from total PXO99^{A} RNA.

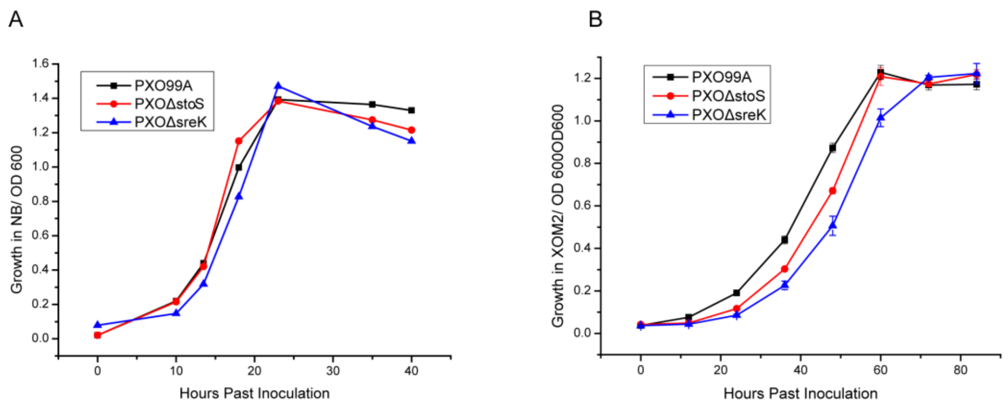

Figure S2. Growth curve of *PXOΔstoS*, *PXOΔsreK* and PXO99^{A}. *Xoo* strains were activated in 5ml NB medium for 24-36 hours at 28℃. The activated cultures which were adjusted to OD_{600} 1.0 were then 1/100 inoculated to 100ml NB medium (A). To measure the growth in XOM2, NB medium in the activated cultures were removed, and the bacteria pellets were resuspended and adjusted to OD_{600} 1.0 in XOM2. The adjusted cultures were 2/100 inoculated to 100ml XOM2 medium (B). The absorbance at 600nm of the cultures was measured at certain time.

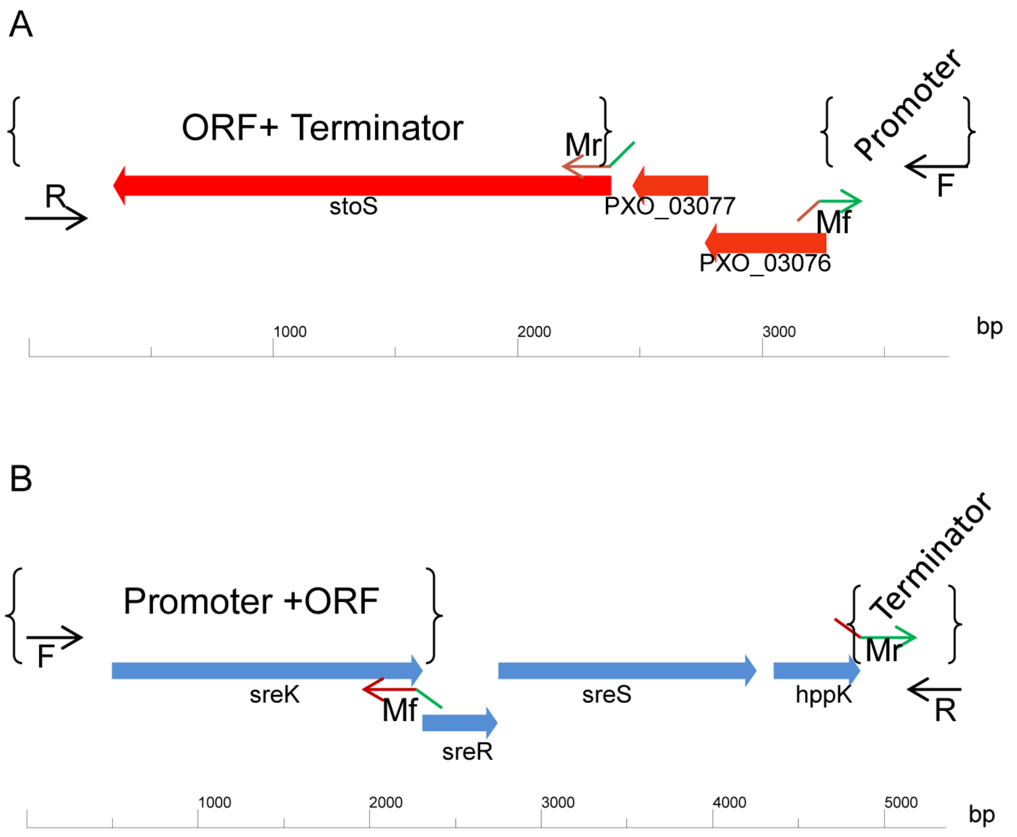

Figure S3. Gene complementation strategy for *stoS* (A) and *sreK* (B). Two DNA segments were got using F+Mf and R+Mr as primer pairs. The two DNA segments were ligated together via overlapping PCR using F+R as the primer pairs. The full length of the ORF together with its native promoter and terminator were subsequently cloned to pHM1 by the restriction endonuclease site additionally added in the 5' terminal of primer F and R.

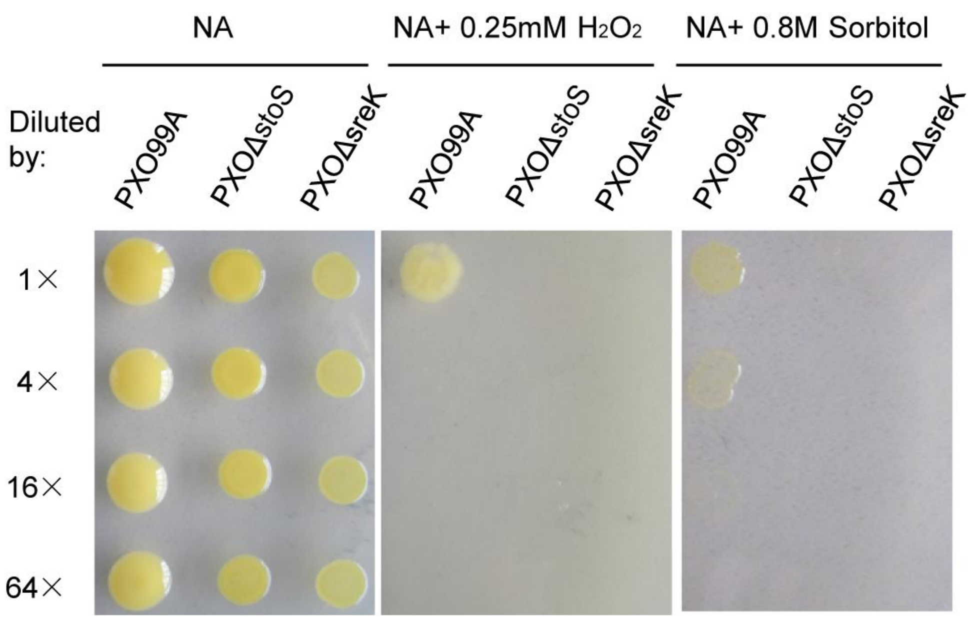

Figure S4. Resistance to activated oxygen and osmotic pressure assays. *Xoo* strains were grown in NB medium and adjusted to OD_{600} = 1.0 followed by dilutions of four-, 16-, and 64-fold. Serial dilution cultures (2 μL) were spotted on NA medium containing 0.25 mM H_2O_2 to determine the resistance to reactive oxygen. Osmotic pressure assays were performed on 0.8 M sorbitol added NA medium. All cultures were grown at 28 °C for 48 to 72 hours to analyse the result.
